# Development of a Fall-Specific Impact Testing Method to Evaluate Safety Helmet Performance and Injury Risk

**DOI:** 10.1007/s10439-025-03930-9

**Published:** 2025-12-09

**Authors:** Susanna M. Gagliardi, Nicole E.-P. Stark, Mark T. Begonia, Michael L. Madigan, Steven Rowson

**Affiliations:** 1 Department of Biomedical Engineering and Mechanics, Virginia Tech, 325 Stanger St., Kelly Hall 120, Blacksburg, VA 24061 USA; 2 Institute for Critical Technology and Applied Science, Virginia Tech, Blacksburg, VA USA; 3 Department of Industrial and Systems Engineering, Virginia Tech, Blacksburg, VA USA

**Keywords:** Hard hat, Skull fracture, Concussion, Risk, Falls, Oblique, Head acceleration, Rotational, Construction

## Abstract

**Purpose:**

Despite falls accounting for the greatest number of fatal and non-fatal work-related traumatic brain injuries, current standards do not evaluate safety helmets under impact conditions representative of fall scenarios. This study’s objective was to develop a test method that evaluates safety helmets under impact scenarios representative of falls. A Construction STAR rating system that quantitatively compares safety helmet performance in the context of concussion and skull fracture risk is also outlined.

**Methods:**

A multi-step approach that combined information from previous literature, Occupational Safety and Health Administration (OSHA) accident reports, and oblique impact tests were used to develop a fall-specific safety helmet test methodology. The test methodology consisting of three impact locations (front boss, rear boss, and rear), two impact velocities (5.5 and 6.8 m/s), and a 25-degree anvil was executed on a representative subset of one Type I and four Type II models. STAR scores, combining concussion and skull fracture risk, were calculated for each model and compared.

**Results:**

STAR scores demonstrated that Type II helmets reduced concussion risk by 32.7% and skull fracture risk by 57.5% when compared to the Type I model. Large variations in Type II performance were observed, with the top-performing Type II helmets reducing concussion risk by 28.7 and 33.2% compared to bottom-performing models.

**Conclusions:**

Type II helmets offer substantial benefits in head protection compared to Type I models for oblique fall-related impacts. By including both skull fracture and concussion risk in the STAR score, the proposed methodology can differentiate high and low-performing safety helmets.

## Introduction

Concussion awareness has become increasingly popular in sport settings, with numerous studies highlighting the importance of helmets or headgear to reduce concussion risk [1–9]. While sport concussion awareness has resulted in more effective helmet designs and improved head protection, less emphasis has been placed on occupational head injuries and safety helmet design advancements [10–14]. Work-related traumatic brain injuries (WRTBIs) are estimated to represent 4–24% of all traumatic brain injuries (TBIs) reported each year, while sport-related TBIs in adolescents are estimated at 10%, highlighting the prevalence of occupational head injuries [15–17]. Additionally, individuals in construction or industrial settings face greater exposure to head injuries due to prolonged work in high-risk environments, as opposed to isolated practices or games typical in sport settings. Due to these risk factors, head injury severity is also greater in construction settings, with the incidence of skull fracture and moderate-to-severe TBIs being higher than the incidence of mild TBIs [18, 19]. As a result, WRTBIs are associated with serious socioeconomic implications, including medical costs, loss of work, and the potential for long-term disability, all of which severely impact quality of life [17, 18, 20]. Given these challenges, TBI and skull fracture prevention in workplace settings is critical.

Across all sectors, the construction industry accounts for the largest number of fatal and non-fatal TBIs due to various environmental risks associated with the profession, including working at height, falling objects, and heavy machinery [18, 21–23]. Falls are the leading cause of WRTBI [24–27], with falls from elevation representing a particular concern in the construction industry [22, 28–30]. Particularly, 54% of non-fatal TBIs and 57% of fatal TBIs in the construction industry are due to falls, emphasizing the need for personal protective equipment (PPE) that protects against fall injury scenarios [26, 27]. According to current Occupational Safety and Health Administration (OSHA) standards, workers must wear head protection when exposed to head impact hazards and employ fall protection when working at elevations exceeding 6 ft in the construction industry [31, 32]. Despite these regulations, failure to use fall protection is consistently the most common OSHA violation, leaving safety helmets as the only protective measure for many workers [33]. Safety helmets (also referred to as hard hats) are used in industrial settings as a form of head PPE. In ANSI Z89.1-2014 a helmet is defined as “A device worn on the head designed to provide limited protection against impact, flying particles, or electrical shock [23].” Safety helmets are classified under two categories by the U.S. safety helmet standard ANSI Z89.1-2014 (R2019): Type I and Type II [34]. Type I helmets are tested for impacts to the top of the head, whereas Type II helmets are tested for impacts to the top and side [34]. To receive certification, industrial head protection must meet the ANSI/ISEA Z89.1-2014 testing standards, which cover requirements for flammability, force transmission, apex penetration, and electrical insulation [34]. These standards do not include tests for the explicit purpose of protecting the user against fall-induced head impacts, although they are the largest cause of fatal WRTBIs [22, 28–30]. Although safety helmets are required on all construction/industrial sites where there is a risk of head impact, the helmet certification standards are based on impact energies that are too low to represent typical fall scenarios [13, 35]. Moreover, ANSI Z89.1-2014 (R2019) entails various vertical drop tests onto a helmeted headform, which constrains the headform and test systems to linear motion [34]. As a result, only linear acceleration is evaluated during these standard tests, despite rotational head motion being observed in real-world head impacts [36–38]. While early research supported the idea of linear acceleration being the primary predictor for concussion, recent research has demonstrated that rotation also contributes to concussion risk through producing shear strain in neural tissues [39–41]. Because approximately 30% of non-fatal WRTBIs result in a concussion, evaluating safety helmets for their ability to mitigate concussion risk is of value [21].

Recent work on industrial safety helmet performance spans both ANSI-style drop tests and more realistic fall/trip simulations. Two studies using headforms subjected to object drops modeled on ANSI Z89.1-2014 reported that safety helmets and hard hats reduced head acceleration relative to an unprotected condition [14, 42]. To capture worker kinematics during falls, Yu et al., evaluated a free-fall headform without a Hybrid III neck across four representative fall scenarios onto 45° and 15° anvils at 2.7–5.5 m/s [43]. Complementing this, Bottlang et al. assessed head protection using a guided drop tower with a Hybrid III neck onto a 30° anvil at 3.5 and 6.2 m/s [13]. Taken together, these studies indicate that industrial helmets attenuate head loads under both normal and oblique impacts; however, differences in test protocols (e.g., neck inclusion, anvil angle, and impact speed) complicate direct comparisons and underscore the need for evaluations that replicate realistic fall dynamics.

Testing safety helmets under realistic head impact conditions is essential for assessing concussion risk, however, establishing accurate boundary conditions to replicate fall-related head impacts in a laboratory setting remains a challenge. Falls in construction often occur on unfinished job sites, resulting in a limited number of falls being captured on video. Additionally, if video is captured, it is often not publicly shared to protect the privacy of the individual(s) involved and the organizations associated with the accident. While survey data and accident reports filed by OSHA document information such as fall surface, fall height, injuries sustained, and impact surface, crucial information necessary for fall reconstruction in laboratory settings, including head impact location, angle, and velocity, is not typically reported [44]. As a result, numerous studies have attempted to model falls using computer simulations based on subject interviews, fall videos, or laboratory human subject data that arrested the fall before any impact with the ground [45–51]. However, computer models are limited as they may not accurately capture human fall arrest movements that help lower the fall energy, known as bracing [52–54]. While bracing can only be observed in human subject laboratory testing, there are a limited number of laboratory studies that explore falls from height due to concerns regarding subject safety [52]. Additionally, there are other fall impact mitigating mechanisms (FIMMs) such as accidental contact with objects during the fall, air resistance, and other body parts impacting the ground first that are not represented in computer simulations. Nonetheless, previously published laboratory and computational studies provide an estimate for common head impact angles, locations, and velocities associated with falls from height in the absence of more direct data.

The Summation of Tests for the Analysis of Risk (STAR) assessment method was originally developed to supplement helmet standards testing and provide a quantitative comparison of impact performance between helmet models [55]. STAR methodologies have been developed for several sports including varsity football [55], youth football [2, 56, 57], flag football [58], hockey [1], bicycle [3, 59, 60], soccer [61, 62], snow sports [63, 64], whitewater [65, 66], and equestrian [67, 68]. The development of the STAR methodologies are based on two fundamental principles. First, a series of field-driven laboratory tests is conducted, where each impact scenario is weighted by its frequency of occurrence in the real-world. Second, helmets that mitigate rotational and linear head kinematics decrease the risk of concussion. Evidence suggests that STAR has contributed to the development of safer helmet designs. In NCAA football cohorts, players wearing higher-rated helmets sustained fewer concussions than those wearing lower-rated models [11, 69]. Since the introduction of STAR for bicycle helmets, manufacturers have significantly lowered rotational kinematics compared to early models. Modern football helmets have also shown notably reduced linear and rotational accelerations compared to those rated in the initial football STAR release. These combined effects reflect broad design improvements aligned with STAR guidance. Although similar epidemiologic validation is not yet available for construction helmets, these cross-category trends support the relevance of the STAR framework to be applied to industrial head protection. However, in the construction industry, catastrophic head injuries such as skull fractures are also of interest, as they are common along with mild/severe WRTBI. One study estimated that construction-related skull fractures and concussions have an incidence rate of 65.8 and 47.1 per 100,000 workers, respectively, highlighting the prevalence of both injury types [18]. As such, a STAR evaluation method for safety helmets in construction should consider both skull fracture and concussion.

This study aims to develop a STAR testing methodology for safety helmets that includes impact conditions representative of real-world fall scenarios. Through analyzing accident report data, performing oblique impact testing, and examining previously published literature, the necessary boundary conditions to recreate fall-related head impacts in a laboratory setting can be identified. A rating scheme that creates a comprehensive STAR score describing the helmet’s ability to mitigate concussion and skull fracture risk under these fall scenarios is also outlined. This methodology can serve as a guideline for helmet manufacturers to design safety helmets that perform well in severe but survivable fall scenarios. Safety helmets that are tested and perform well under realistic impact conditions have the potential to reduce the number of fall-related skull fracture and TBIs in industrial settings. Moreover, a rating scale enabling quantitative comparisons among various safety helmets will inform consumers about the most protective models available on the market.

## Methods

A multi-step approach that examined accident reports, previous literature, and oblique impact tests was used to identify boundary conditions representative of fall scenarios in construction. The OSHA Fatality and Catastrophe Investigation Summaries database was searched to understand common fall scenarios in construction. Normal head impact velocities representative of 50th and 85th percentile fall heights, as determined from OSHA data, were then used to estimate the normal head impact velocity using an analytical approach that accounts for head impact energy reductions due to FIMMs. Previous literature, consisting of computational modeling studies and induced human subject ladder falls, allowed us to estimate the range of tangential head impact velocities as well as head impact locations. Using the target normal and tangential head impact velocities, an anvil angle for oblique impact testing was determined. Oblique tests using the aforementioned impact conditions were then performed on sandpaper and a tile surface representative of concrete to determine the appropriate impact surface. Lastly, a STAR testing methodology combining the previous work was applied to a representative subset of five independent safety helmet models, and a rating scheme was developed to quantitatively evaluate helmets for their ability to mitigate concussion and skull fracture risk.

### OSHA Accident Report Search

Common fall scenarios were identified by searching the OSHA Fatality and Catastrophe Investigation Summaries database, which reports on fatal and catastrophic events. The search terms “fall AND concussion”, “fall AND brain”, and “fall AND skull” were used to identify falls with head injuries between the dates of January 1st, 2009, to September 30th, 2024. The search was restricted to North American Industry Classification job codes 236, 237, and 238, which correspond to construction of buildings, heavy and civil engineering construction, and specialty trade contractors, respectively. For each accident report, the fall height (measured from the feet), injuries sustained, fall surface, impact surface, fatality, age, sex, and head impact location were recorded when available. Accident reports varied in the level of description, resulting in some variables not being documented for every entry. Injuries were broadly categorized into four groups: concussion, skull fracture, brain hemorrhage, and unspecified head injuries. While a given accident report could result in a combination of the injuries outlined, unspecified head injuries denoted that the accident report was not detailed enough to definitively determine a specific injury, but that the individual sustained a fall-related head injury. As such, if an unspecified head injury was recorded for an accident report, no other injury groups were documented. The frequency and proportion of impact surfaces, as well as the distribution of fall heights, were used in subsequent analyses to determine the appropriate laboratory impact conditions.

### Normal Head Impact Velocity Determination

While common fall heights in construction are well-understood, a direct association between fall height and head impact velocity is unknown due to FIMMs, which account for the voluntary and involuntary actions that lower the head impact severity during a fall. Examples of FIMMs include intentional movements during the airborne phase of a fall or upon impact that alter the body part(s) impacting the ground, unintentionally allowing other body parts to impact the ground before the head, and/or contacting objects during the fall. To attempt to estimate head impact velocity in the presence of FIMMs, an analytical approach that used real-world skull fracture data from falls and previously published skull fracture risk curves was used [70]. Through matching skull fracture risk between real-world fall heights and postmortem human subjects (PMHS) with laboratory drop tests, we could relate real-world fall heights to lab drop speeds. This was done through relating the PLA or head injury criterion (HIC) that corresponded head impact velocities determined from a series of laboratory drop tests onto a bare and helmeted NOCSAE headform. The skull fracture risk associated with this PLA/HIC values from bare head impacts was determined through association with published PMHS head impacts. Then, the fall height corresponding to skull fracture risk for helmeted impacts was determined using a risk curve created from OSHA accident reports. On average, FIMMs reduced a person’s worst-case fall velocity (i.e., head-first fall) by 44.5–64.4%. This analysis was then used to determine the normal drop speeds representative of a 50th and 85th percentile fall heights determined from the OSHA accident report analysis.

### Anvil Angle and Tangential Head Impact Velocity Determination

While normal velocity was determined using an analytical approach, previously published literature, including both human subjects and computer modeling studies, was evaluated to determine a realistic range of tangential head impact velocities during fall scenarios. First, a study by Ferro et al. that included human subject ladder falls while capturing whole-body kinematics was examined [52]. This study had 15 total head impacts from falls ranging from 0.8 to 1.8 m, as measured from the feet. The tangential velocities associated with these falls were calculated as the resultant of the anterior-posterior and medial-lateral velocity components, as defined in the study [52]. Second, a study by Yu et al. that performed 1692 simulations of fall and trip events using a multi-body human model (MADYMO) from various heights and body orientations was analyzed [50]. This study modeled trips, forward falls, and backward falls while altering worker size, initial posture, fall height, walking speed, and tripping barrier dimensions to obtain a representative average of head impact speeds, angles, and locations during various industrial head impact events [50]. Using their suggested impact speed (v_resultant_) and angles (*θ*) for the fall conditions, the tangential velocity was computed. Using the normal velocity components outlined in the previous section, the impact angle was selected to give tangential velocities within the range of Ferro et al. and Yu et al. This impact angle was used to decide the anvil angle for all subsequent impact testing.

### Location Determination

A combination of previous literature and a subset of surveys detailing real-world industrial head impacts was analyzed to select the appropriate impact locations for safety helmet testing. First, the Ferro et al. study was examined, as it gave a visualization of head impact locations for all 15 ladder falls (5 sideway falls, 10 backward falls) observed in their study [52]. Second, a study by Proctor and Rowland, which outlined two different surveys examining industrial head injury locations, was considered. Lastly, the azimuth and elevation angles reported during simulated fall events from Yu et al. were examined to gain a better understanding of possible head impact locations for forward and backward fall scenarios [50].

Along with the literature review, a custom survey was designed to gather detailed information on head impact locations during industrial head impacts, supplementing information from the OSHA accident report search (VT IRB #24-972). Any industrial worker who experienced or nearly experienced a head impact while working in an industrial setting was included. For this analysis, only responses that involved falls to a lower level were considered. Respondents were asked to document the location of their head impact based on Fig. 8. These responses, combined with the literature review, were used to select the appropriate locations for safety helmet impact testing.

### Impact Surface Testing

Using the OSHA accident report analysis data, the frequency and proportion of impact surfaces were determined. Friction testing for the most common impact surface (concrete) was performed using a standard sled test against three different common helmet shell materials: acrylonitrile butadiene styrene (ABS), high-density polyethylene (HDPE), and polycarbonate (PC). A concrete slab was rigidly adhered to a table using clamps. A 20.98 kg sled with flat samples of the helmet shell material on its bottom was placed on top of the concrete slab. One end of a steel cable was rigidly attached to the sled, while the other end was attached to a bucket. A pulley was placed at the edge of the table, and the cable connecting the sled and bucket was laid over the pulley, allowing the bucket to hang freely over the edge of the table. The weight of the bucket was incrementally increased by 0.20 g until movement was generated. The bucket weight that first generated movement was recorded. Three trials per helmet shell material were conducted, and helmet shell material samples were replaced after every test. The static coefficient of friction (COF) of the helmet shell material on the concrete was calculated using Eq. 1, where *m*_*bucket*_ and *m*_*sled*_ are the mass of the bucket and sled, respectively.

1 $$\mu =\frac{{m}_{bucket}}{{m}_{sled}}$$

To determine the COF of helmet shell materials on other materials that can be used during impact testing, a similar procedure was followed with the concrete slab being replaced by 80-grit sandpaper and commercial floor tile. 80-grit sandpaper was chosen as it is commonly used in oblique helmet testing and commercial floor tile was chosen to try and match the COF of the concrete slab. Both materials were selected due to enhanced repeatability and ease of use when interchanging the impactor face.

**Fig. 1:**
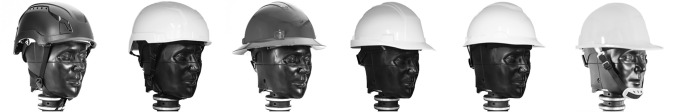
All helmet models tested, from left to right: PIP Traverse MIPS, Milwaukee Tool BOLT, ERB Safety Americana 360, Honeywell Matterhorn A89, 3 M SecureFit, and UNLINE Standard Hard Hat. The 3 M SecureFit and UNLINE Standard Hard Hat are Type I helmets

While the static friction tests were used to compare the COFs of 80 grit sandpaper, commercial floor tile, and concrete on different helmet shell materials, surface friction during impact testing was further investigated. For this test series, a representative subset of five safety helmet models were examined, consisting of four Type II models (PIP Traverse MIPS, Honeywell Matterhorn A89, ERB Safety Americana 360, Milwaukee Tool BOLT and one Type I model (3 M SecureFit) (Fig. 1).

### Headform Selection, Constraints, and Helmet Installation

A 50th-percentile National Operating Committee on Standards for Athletic Equipment (NOCSAE) headform was used for all impacts. Developed specifically for helmet testing, the NOCSAE headform has a more biofidelic shape than the Hybrid III headform [72–74]. Comparatively, the Hybrid III was originally designed for automotive crash testing and, although it is used in some helmet evaluations [75–79]. The Hybrid III also exhibits fidelity shortcomings relative to the human head, further motivating our choice [80, 81]. There are moment of inertia (MOI) differences that should be considered [81, 82]. Moreover, because the NOCSAE headform was designed with helmet testing in mind, it better replicates realistic helmet fit and contact conditions, leading to more accurate assessment of helmet performance [72, 83, 84].

**Fig. 2:**
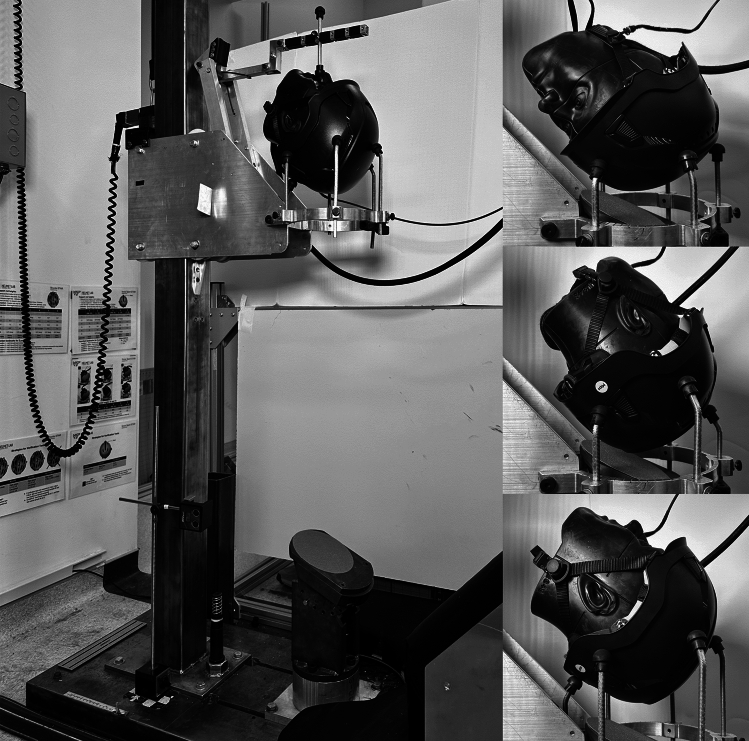
The oblique drop tower used for safety helmet testing. For the impact surface determination test series, the anvil surface alternated between commercial floor tile and 80-grit sandpaper. The front boss (top), rear boss (middle), and rear (bottom) impact locations were selected for impact testing

The NOCSAE headform was tested on an oblique drop tower without an attached neck (Fig. 2). Analytical considerations and prior studies show that fall-related head impacts contain both normal and tangential velocity components, which the oblique tower reproduces; our kinematic traces are consistent with those reported for head-to-ground impacts [77, 85]. The exclusion of the neck was based on several factors. The Hybrid III neck was originally developed for the automotive sector and validated by comparing its flexion and extension responses to those from post-mortem human surrogates (PMHS) during frontal sled tests [86]. Despite its wide usage, the HIII neck has been shown to be less biofidelic in other loading scenarios and directions, especially axial loading [78, 87–92] though the presence a neck has been debated [78, 82, 87, 92–94]. Due to the rigidity of the neck and impact angles, inclusion of the neck would deviate further away from a biofidelic response than not including a neck at all. Similar approaches have been used extensively in helmet testing [80, 95–97].Consistent with this, guided drop tower comparisons with and without a Hybrid III neck showed that including the neck produced secondary peaks in rotational velocity and acceleration not seen in other ATD helmet-to-ground tests [76, 77, 82].

All helmets were positioned 2.5 cm above the NOCSAE headform brow line using a custom-built nose gauge. The retention system of the helmet was adjusted until slight resistance was met, and retention straps were tightened until nearly taut under the headform. If no retention straps were present, a small piece of painter’s tape was placed on the helmet/headform near the headform’s temples. This ensured that the helmet stayed on during positioning but was not unrealistically/rigidly adhered to the headform upon impact. The centeredness of the helmet was checked using the headform’s mid-sagittal anatomical plane.

### Helmet Testing

A helmeted 50th percentile male NOCSAE headform was dropped onto a 25° steel anvil covered with 80-grit sandpaper or commercial floor tile. Sandpaper was replaced after every fourth test for consistency and floor tile was replaced after every twelve tests, as it did not wear as frequently as the sandpaper. The helmeted headform was supported using a support ring attached to the drop tower and an additional support arm that mechanically releases before impact.

Helmets were impacted at three locations (front boss, rear boss, and rear) as determined by the impact location research (Fig. 2, Table 3). To ensure consistent headform and helmet positioning, a dual-axis inclinometer (WT9011DCL-BT50, WitMotion, Shenzhen, China) was mounted parallel to the base of the headform to display the headform X and Y orientation. Orientation from the dual-axis inclinometer was constrained to be within 0.5° of the desired placements. To specify the Z rotation, a cross-line laser was aligned with the midsagittal and transverse planes marked on the NOCSAE headform and projected onto the support ring, inscribed with 5° increments. The projected Z angle was constrained to be within 2.5° of the desired angles. The helmet positioning process is described in detail in the testing methodology open-access documentation [98]. One trial per impact condition was conducted across all five helmet models, and a new helmet sample was used for each impact test to prevent helmet degradation. If a low-speed impact at a given location resulted in a PLA above 350 g, the high-speed test was not conducted.

The NOCSAE headform was instrumented with a 6-degree-of-freedom sensor package containing three accelerometers and three angular rate sensors placed at the headform’s CG (Endevco 7264B-2000, PCB Piezotronics, Depew, NY) (ARS3 PRO-18 K, DTS, Seal Beach, CA). Data were sampled at 20 kHz and filtered using a 4-pole Butterworth lowpass filter with a cutoff frequency of 1650 Hz for accelerometer data and 300 Hz for angular rate sensor data. Angular acceleration was obtained by differentiating the angular velocity data using a five-point central difference method, as outlined in SAE J211 [99]. The resultant PLA and peak rotational acceleration (PRA) were reported for each impact test.

### STAR Testing Methodology

After identifying the impact testing conditions representative of fall scenarios, a test methodology was developed and executed on five independent helmet models (Fig. 1). This included four Type II models (PIP Traverse MIPS, Honeywell Matterhorn A89, ERB Safety Americana 360, Milwaukee Tool BOLT and one Type I model (UNLINE Standard Hard Hat). The same test oblique impact setup and methodology were used as previously outlined, on a single impact surface (Fig. 2). Impact tests were performed at three locations (front boss, rear boss, rear) and two energy levels (5.5 m/s and 6.8 m/s) (Fig. 2). A 25° steel anvil covered with 80-grit sandpaper was used for all impact tests, and the sandpaper was replaced after every fourth test. Two trials were conducted per impact condition, for repeatability, where a new helmet sample was used for each test, totaling 12 tests per helmet model. We assessed repeatability using a 15% tolerance criterion on the range-over-mean of PLA across repeated trials; if any trial exceeded this 15% threshold, a third verification trial was performed. Impact tests were not replicated if the initial trial produced a PLA greater than 350 g. Additionally, if a low-speed impact at a given location resulted in a PLA above 350 g, the corresponding high-speed impact was not conducted. This limit was used to avoid potential damage to the test equipment and because concussion and skull fracture risk functions are effectively saturated (≥ 99% risk) above this level. Data were filtered as previously outlined, and peak head kinematics (PLA and PRA) were recorded for each impact and averaged across trials for the same impact condition. Concussion risk was calculated for each impact condition using a previously established bivariate risk function (Eq. 2) where $a$ is the magnitude of the three-dimensional resultant peak linear acceleration and $\alpha$ is the magnitude of the three-dimensional resultant peak rotational acceleration [36]. This risk function was derived using data that was collected from collegiate male football players who wore helmets instrumented with sensors and then paired with diagnosed concussions [36].

2 $${R}_{brain}\left(a,\alpha \right)=\frac{1}{1+\mathrm{exp}\left(-\left(-10.2+0.0433a+0.000873\alpha -0.000000920a\alpha \right)\right)}$$

Due to the prevalence of skull fracture injuries both from the literature and observed in the OSHA accident report analysis, all safety helmet tests were also evaluated for skull fracture risk [18, 28]. Skull fracture risk was calculated using the PLA from each impact condition from a previously established risk curve based on peak head acceleration [100]. This risk curve uses a normal cumulative distribution function (Eq. 3) with a mean (*µ*) of 262 g and a standard deviation of (*σ*) of 48 g. The mean, standard deviation, and PLA are used as inputs into the error function (erf), as described by Eq. 3 (R Stats Package, pnorm).

3 $${R}_{skull}(a)=\frac{1}{2}\left[1+\mathrm{erf}\left(\frac{a-\mu }{\sigma \sqrt{2}}\right)\right]$$

A Construction STAR score was calculated for each helmet model to summarize helmet performance into a single value (Eq. 4). The exposure term, *E*, is a function of impact speed, with equal weighting applied across impact locations. The exposure weights were determined by the frequency observed in real-world scenarios. All low-speed impacts were given an exposure value of 22.5, and high-speed impacts were given an exposure value of 10.83. This was chosen since the low-speed impacts represented a 50th percentile fall height, and the high-speed impacts represented an 85th percentile fall height. The midpoint between those two percentiles was used to create two bins (67.5 and 32.5% of impacts), which were then divided evenly between the three locations, resulting in the previously stated exposure values. While exposure values were dependent on impact speed (*V*), they were equally weighted across locations. For each impact configuration, concussion risk (${R}_{brain}\left(a,\alpha \right)$) (Eq. 2) and skull fracture risk (${R}_{skull}(a)$) (Eq. 3) were calculated using the average PLA and PRA and then multiplied by the respective exposure value. If a helmet model exceeded the 350 g peak linear acceleration (PLA) threshold during low-speed testing and was therefore not tested at high speed, both skull fracture and concussion risks were assumed to be 100% for the high-speed impact condition. Weighted risks were then summed to give each helmet model a Construction STAR score that estimates the combined incidence of concussion and skull fracture given 100 head impacts. Since a single head impact can result in a concussion and skull fracture (counting as two separate injuries), the combined incidence represents the total number of head injuries out of 200 potential head injuries (100 concussions, 100 skull fractures).

4 $$ConstructionSTAR= \sum_{L=1}^{3}\sum_{V=1}^{2}\left[E\left(L,V\right)*{R}_{brain}\left(a,\alpha \right)+E\left(L,V\right)*{R}_{skull}\left(a\right)\right]$$

### Statistical Analysis

To evaluate surface friction, linear mixed effects models (lmerTest Package) with helmet model as a random effect was conducted in R (Version 4.4.0, RStudio; Boston, Massachusetts, USA) to quantify the effects of impact speed, location, and surface on PLA, PRA, concussion risk, and skull fracture risk [101]. Linear mixed effects models were also conducted to quantify the effects of impact speed and location on PLA, PRA, concussion risk, and skull fracture with the helmet model as a random effect. A significance level of *α* < 0.05 was selected, and post hoc comparisons were completed using least squares means (lmerTest Package) [101]. When performing this analysis, the 3 M SecureFit helmet was removed, as it was the only Type I helmet and was considered an outlier in the dataset.

## Results

### OSHA Accident Report Search

**Fig. 3:**
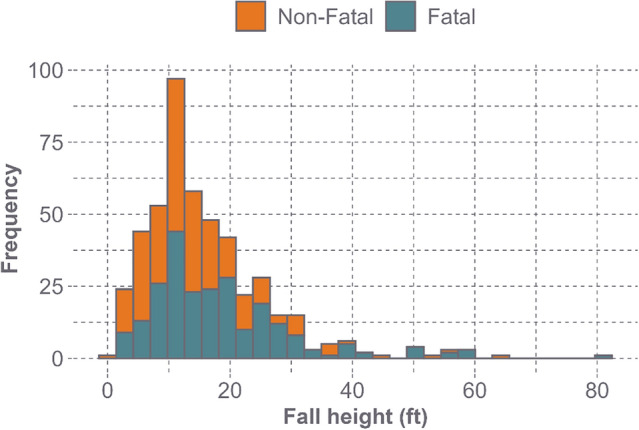
The distribution of fall heights from the OSHA Fatality and Catastrophe Investigation Summaries report search, including both fatal and non-fatal but catastrophic fall scenarios

Given the previously stated search criteria, a total of 591 accident reports were identified, including 301 fatal and 290 non-fatal but catastrophic cases. Fall heights, as measured from the individual’s feet, ranged from 1 to 82 ft, with an average fall height of 16.2 ft (95% confidence interval [CI] 15.3–17.0 ft) and a median of 14 ft (Fig. 3). Ladders were the most common surface individuals fell from, accounting for 24.7% of all falls (*n* = 146), followed by roofs (18.4%, *n* = 109), and openings (13.7%, *n* = 81). When the impact surface was documented (*n* = 267), concrete was the most common impact surface, accounting for 73.0% of the documented impacts (*n* = 195). From the injury data, skull fractures were the most common head injury (50.2%, *n* = 319), followed by unspecified head injuries (25.5%, *n* = 162), brain hemorrhages (12.6%, *n* = 80), and concussions (11.8%, *n* = 75). Since an individual could sustain multiple injuries during one accident event (e.g. skull fracture and brain hemorrhage), the total number of injuries is greater than the number of accident reports analyzed.

### Normal Head Impact Velocity Determination

From the OSHA accident report data, the fall height percentile groups were identified and presented in Table 1. The corresponding normal head impact velocities for these fall heights were then calculated, taking into account the reduction in fall energy due to FIMMs (Table 1).

**Table 1:** Quantile groups for fall heights from the OSHA dataset, with the corresponding normal head impact velocities needed to replicate the fall scenarios in a laboratory setting

Percentile fall height	Fall height (ft)		Fall height (m)	Normal head impact velocity (m/s)
50th	14	4.3		4.9
85th	25	7.6		6.2

### Anvil Angle and Tangential Head Impact Velocity Determination

When observing head impact velocities from real-world ladder falls, Ferro et al. reported tangential head impact velocities that ranged from 1.0 to 4.92 m/s, with a mean of 2.27 m/s [52]. In their suggested impact conditions for forward falls at two impact velocities and backward falls at one impact velocity, Yu et al. reported tangential head impact velocities ranging from 1.42 to 2.69 m/s [50]. When combining these two datasets, the average tangential head impact velocity was 2.20 m/s.

To determine an appropriate anvil angle for impact testing, the tangential head impact velocities for three different anvil angles (20°, 25°, 30°) were evaluated, given the normal impact velocities presented in Table 1. When examining the 20°, 25°, and 30° impact angles, the mean tangential velocities across the two representative fall heights were 2.0, 2.6, and 3.2 m/s, respectively (Table 2). The resultant velocities for the different combinations of normal head impact velocities and anvil angles are also presented (Table 2).

**Table 2:** The tangential and resultant head impact velocities for three different potential anvil/impact angles

	Normal head impact velocity (m/s)	Anvil/impact angle (°)	Tangential head impact velocity (m/s)	Resultant head impact velocity (m/s)
50th	4.9	20	1.8	5.3
85th	6.2	20	2.2	6.6
50th	4.9	25	2.3	5.5
85th	6.2	25	2.9	6.8
50th	4.9	30	2.9	5.7
85th	6.2	30	3.6	7.1

### Location Determination

All ladder falls from the Ferro et al. study resulted in impacts to the rear of the head [52]. Of the 15 head impacts reported in this study, 6 were within 2 cm of the midsagittal plane. When combining the survey responses from a study by Proctor and Rowland, front head impacts accounted for 43.3% of impacts (*n* = 578), followed by the top (26.7%, *n* = 357), the rear (15.3%, *n* = 204), and lastly the side (14.7%, *n* = 196) [71]. While this study provides insight into common head impact locations, the location descriptors were overly generalized, and the head impacts were not restricted to falls. In their proposed methods based on simulated falls, Yu et al. proposed two frontal head impact locations and one rear [50]. The first head impact for a low-speed forward fall resulted in a nearly mid-sagittal forehead impact (− 2.9° azimuth angle, 43.8° elevation), whereas the high-speed forward fall resulted in an off-center frontal impact, lower on the forehead (16.5° azimuth, 38.8° elevation) [50]. Lastly, four survey responses with falls from height were identified (VT IRB #24-972). From these responses, there was 1 impact to the front (Location 1, Fig. 8), 1 impact to the rear (Location 5, Fig. 8), and 2 impacts to the left front boss (Location 8, Fig. 8). Based on this information, three impact locations were selected, including a centered (i.e., along the mid-sagittal plane) rear location, right rear boss, and left front boss (Fig. 2). The corresponding X, Y, and Z angles for each impact location are presented in Table 3.

**Table 3:** The impact locations selected for impact testing, informed by literature and survey responses [36, 37, 40]

Location	X (°)	Y (°)	Z (°)
Rear	0.0	60.0	180
Right rear boss	22.5	35.7	155
Left front boss	− 37.8	− 20.2	− 80

^a^Note that X and Y are defined according to the SAE J211 coordinate system whereas Z is defined by projecting the midsagittal plane of the NOCSAE headform onto the halo. The halo is inscribed in 5° increments, with 0° corresponding to the headform facing the drop tower and positive angles corresponding to a clockwise rotation.

### Impact Surface Determination

The static friction testing found that the average COF of concrete on all helmet shell materials was 0.43 (Table 4). This was noticeably lower than the helmet shell materials on 80-grit sandpaper, which had an average COF of 0.89, and higher than the helmet shell materials on the floor tile, which had an average COF of 0.34.

**Table 4:** The average static COFs [mean ± standard deviation] of the helmet shell materials against various surfaces

Base material	Helmet material	COF
Concrete	PC	0.45 ± 0.01
Concrete	ABS	0.47 ± 0.01
Concrete	HDPE	0.36 ± 0.02
80-grit sandpaper	PC	0.84 ± 0.01
80-grit sandpaper	ABS	0.92 ± 0.01
80-grit sandpaper	HDPE	0.92 ± 0.02
Floor tile	PC	0.46 ± 0.03
Floor tile	ABS	0.37 ± 0.03
Floor tile	HDPE	0.19 ± 0.01

**Fig. 4:**
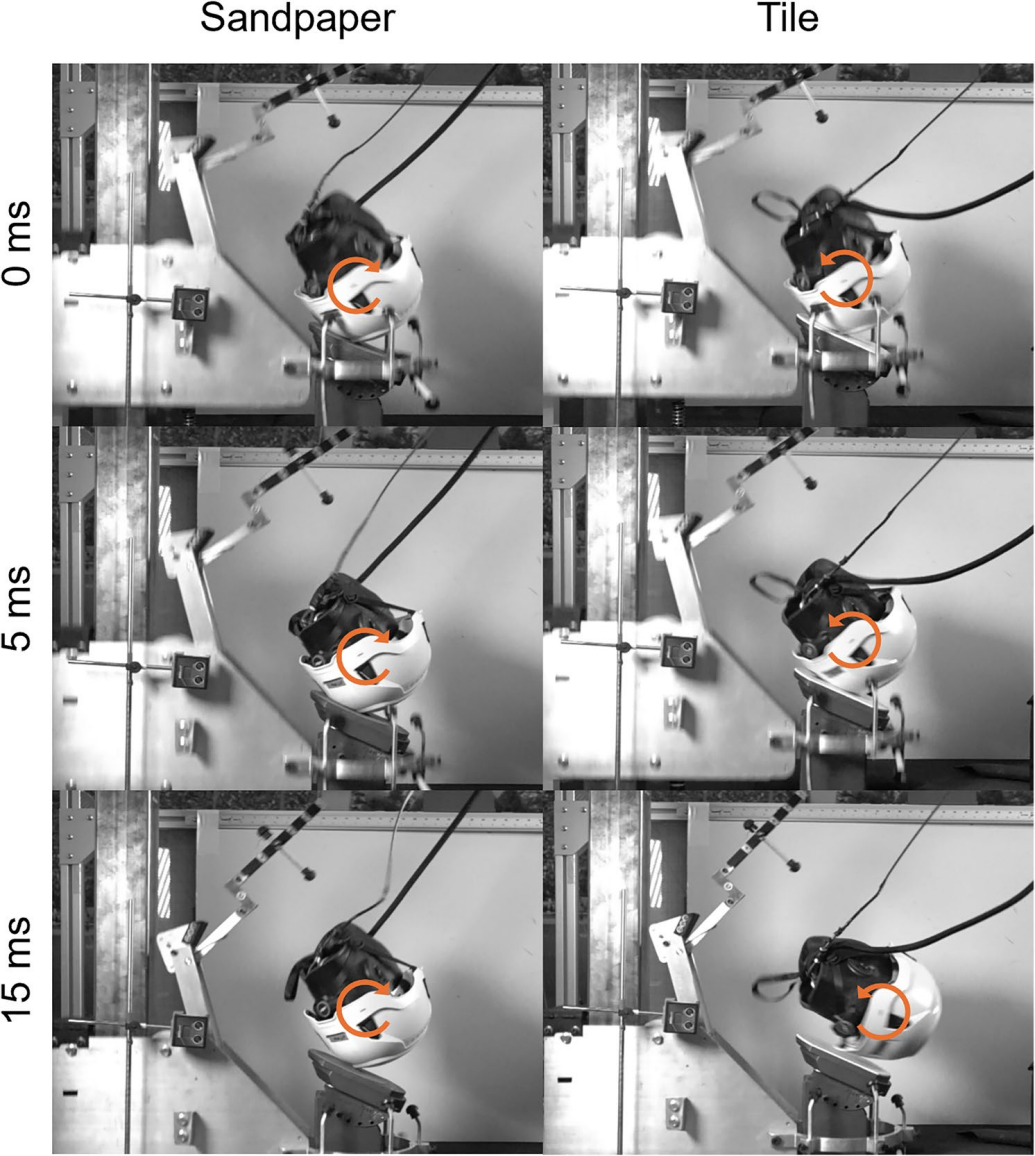
Images from slow-motion video revealed different headform rotations between the sandpaper and tile impact surface conditions, which can be observed in this rear boss impact on the PIP Traverse MIPS helmet. While all sandpaper impacts resulted in clockwise rotation, tile impacts resulted in counterclockwise rotation for some locations and helmet models. The tile impacts resulted in large rotational acceleration values within the same helmet model

Impact testing revealed a large range of peak rotational accelerations both within and across helmet models during the tile condition. Impact location and speed had strong effects on PLA (*p* < 0.001) and PRA (*p* < 0.001). Although on average, tile was associated with 14.9 g lower PLAs than sandpaper, this was not found to be statistically significant (CI − 41.3 to 11.4 g, *p* = 0.271). The tile impact surface had strong effects on PRA and was associated with 2099 rad/s^2^ (CI 878–3320 rad/s^2^, *p* = 0.002) higher PRAs than the sandpaper condition. High-speed video showed differences in headform rotation within the same location and helmet model when comparing the tile and sandpaper conditions (Fig. 4). During the tile impacts, certain locations and helmet models caused the helmeted headform to slip on the anvil and rotate counterclockwise relative to the camera position (Fig. 4). However, the slippage and counterclockwise rotation was not observed during all tile impacts, resulting in a wide range of PRAs within helmet models for the tile condition. In the sandpaper condition, the helmeted headform rotated clockwise after impacting the anvil for all helmet models and locations (Fig. 4).

### STAR Testing Methodology

**Fig. 5:**
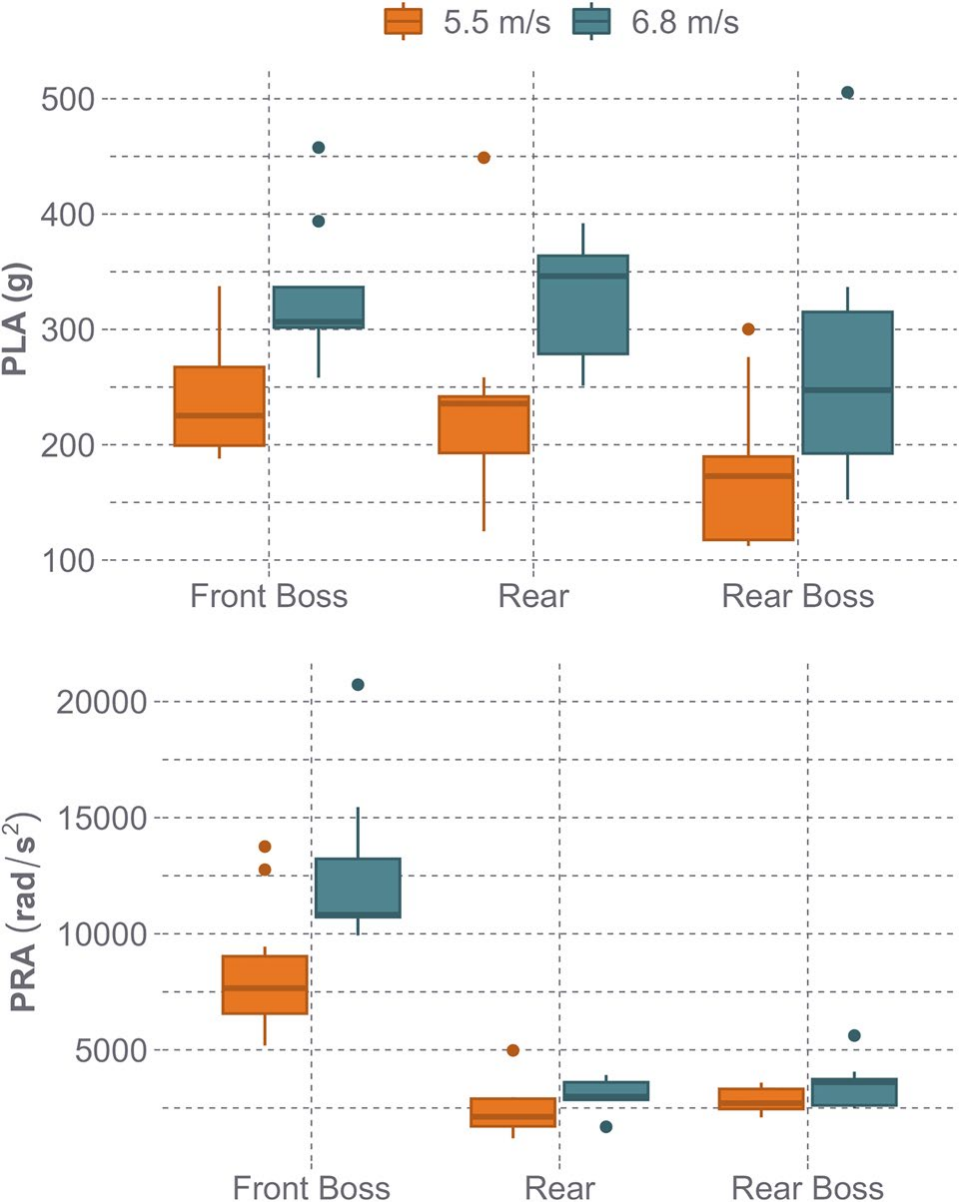
Peak head kinematics for individual impact tests across all helmet models in the STAR test series. Each data point represents a single test. The front boss location reported the highest linear and rotational metrics of all three locations

The peak head kinematics for all helmet models across the three locations and two impact energies are presented in Fig. 5. PLAs ranged from 112.2 to 448.9 g ([mean ± standard deviation] 213.6 ± 74.4 g) for low-speed impacts and 152.4–505.6 g (303.7 ± 86.0 g) for high-speed impacts. PRAs ranged from 1190 to 13757 rad/s^2^ (4590 ± 3296 rad/s^2^) for low-speed impacts and 1688–20729 rad/s^2^ (6786 ± 5103 rad/s^2^) for high-speed impacts. Across all helmet models tested, the mean ± SD of the range-over-mean metric was 6.81 ± 5.08% for PLA and 9.56 ± 9.31% for PRA. The range of PLAs was greater for low-speed impacts as this includes the data from the ULINE Standard Hard Hat. Evaluating Type II models only on average, the 6.8 m/s head impact speed was associated with 100.7 g (CI 72.6–128.8 g, *p* < 0.001) higher PLAs and 2161 rad/s^2^ (CI 1252–3070 rad/s^2^, *p* < 0.001) higher PRAs than the 5.5 m/s condition. The front boss location had lower linear 18.8 g (CI − 54.4.7 to 16.8 g, *p* = 0.305), but higher rotational 7107 rad/s^2^ (CI 5955–8258 rad/s^2^, *p* < 0.001) kinematics compared to the rear location. Additionally, the front boss location was associated with 53.7 g (CI 20.8–86.6 g, *p* = 0.002) higher PLAs and 5938 rad/s^2^ (CI 4886–6990 rad/s^2^, *p* < 0.001) higher PRAs than the rear boss. Due to PLAs above 350 g, five impact conditions did not have a replicate, including: UNLINE Standard Hard Hat rear boss at 6.8 m/s, UNLINE Standard Hard Hat rear at 5.5 m/s, PIP Traverse MIPS front boss at 6.8 m/s, Honeywell Matterhorn A89 rear at 6.8 m/s, and ERB Safety Americana 360 rear at 6.8 m/s.

**Fig. 6:**
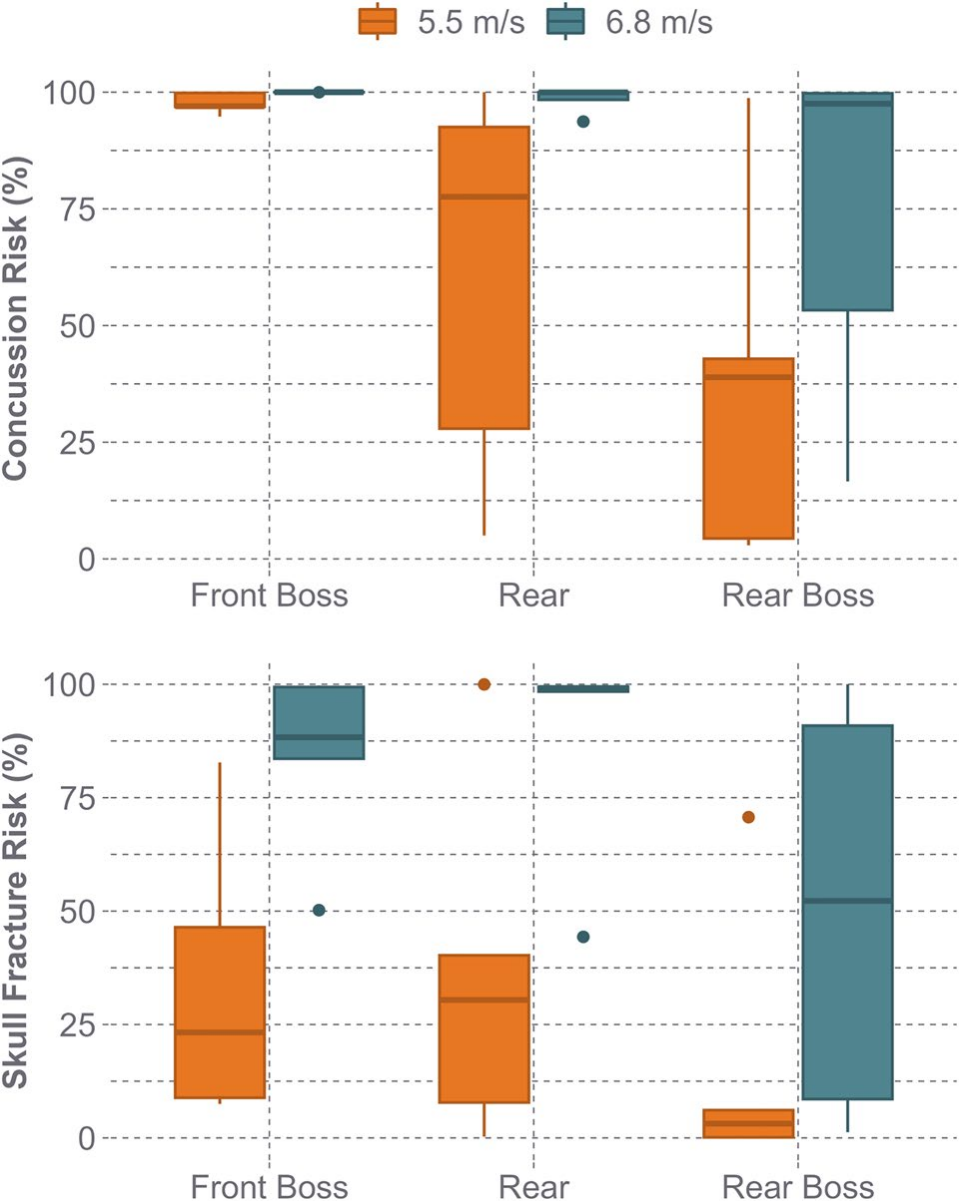
The concussion and skull fracture risk across all helmet models for the STAR test series. There was a wide range of concussion risk across all impact locations at both impact speeds. Skull fracture risk was low at the low-speed condition for most helmet models and was noticeably higher at the high-speed condition

Concussion risk ranged from 2.9 to 100% (65.3 ± 38.6%) at the low-speed condition and 16.6–100% (90.7 ± 22.9%) at the high-speed (Fig. 6). The front boss location reported the highest concussion risk of all locations, and on average had a 24.0% (CI 1.7–46.2%, *p* = 0.049) higher risk than the rear and 54.0% (CI 31.8–76.3%, *p* < 0.001) higher risk than the rear boss location. At high speeds, all helmets at the front boss location had a near 100% risk of concussion. On average, the high-impact speed reported a 31.7% (CI 13.5–49.8%, *p* = 0.003) higher concussion risk than the low speed. Because concussion risk was nearly saturated at the high-impact speed, skull fracture risk was also examined. Skull fracture risk ranged from 0.1 to 100.0% (28.5 ± 31.8%) at the low speed and 1.3–100.0% at the high speed (74.4 ± 31.7%). The 100% risk of skull fracture during low and high-speed tests was due to including the ULINE Standard Hard Hat (Type 1 model), which reported above a 99% risk of skull fracture at four out of the six impact conditions. The highest reported risk of skull fracture at the low speed for helmet models not including the ULINE Standard Hard Hat was 46.4%. The front boss location (51.0%, CI 33.3–68.6%) and the rear location (52.5%, 35.0–70.1%) had the highest skull fracture risk (*p* = 0.898). The rear boss location had the lowest skull fracture risk and had a 30.7% (CI 7.7–53.7%, *p* = 0.018) and 32.2% (CI 9.2–55.2%, *p* = 0.014) lower skull fracture risk than the front and rear locations, respectively. The high-speed condition was associated with a 53.4% (CI 34.7–72.2%, *p* < 0.001) higher skull fracture risk than the low-speed condition.

**Fig. 7:**
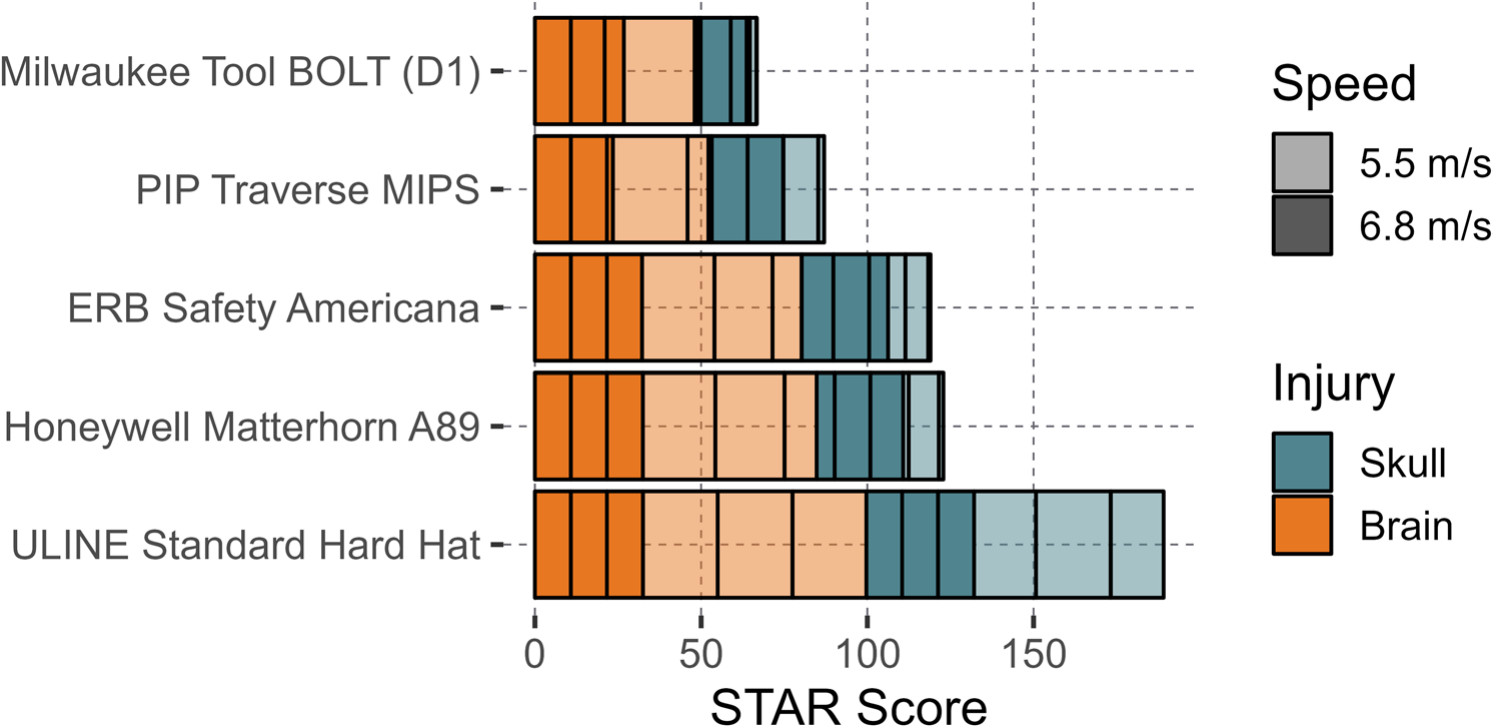
The STAR score for each helmet model tested, including both skull fracture and concussion (brain) incidence

The STAR scores for each helmet model are presented in Fig. 7. The Milwaukee Tool BOLT reported the lowest STAR score (49.8 concussions and 16.9 skull fracture per 100 head impacts), while the UNLINE Standard Hard Hat reported the highest (99.7 concussion and 89.4 skull fracture per 100 head impacts). On average, the Type II helmets reduced concussion risk by 32.7% and skull fracture risk by 57.5% when compared to Type I model. Additionally, large variations in Type II helmet performance were also observed. The top two performing Type II helmets (Milwaukee Tool BOLT and PIP Traverse MIPS reduced concussion risk by 28.7 and 33.2% when compared to the bottom two Type II helmets (ERB Safety Americana 360 and Honeywell Matterhorn A89).

## Discussion

In the absence of direct kinematic data and videos of fall events, previous literature, accident reports, and oblique impact tests were examined to develop a safety helmet test methodology that represents real-world fall scenarios. Our methods build on previous literature by reproducing fall-representative kinematics systematically determined through evaluating OSHA accident reports, prior literature, and experimental testing. Additionally, we quantified both linear and rotational head kinematics and reported injury metrics that enable a direct comparison between safety helmet models. The proposed methodology generated peak head kinematics that distinguished differences in concussion and skull fracture risk across safety helmet models with varying levels of protection.

While the OSHA data presented a large range of fall heights, with one occurring from 82 ft, the average (16.2 ft) and median (14.0 ft) fall heights were much lower. The accident report data found that 41.3% of all falls occurred between 11 and 20 ft, and 23.1% occurred at falls greater than 20 ft. This aligns with a previous large-scale work-related fall survey (493 responses) that found that 31.2% of falls occurred between 11 and 20 ft, and 33.3% occurred at heights above 20 ft [44]. Because of the prevalence of fall heights greater than 11 ft, it was important that fall heights within that range were represented in our laboratory-tested energy levels. This was accomplished through testing at 5.5 and 6.8 m/s, which represented a 14 ft and 25 ft fall, respectively, while accounting for energy reductions due to FIMMs.

In the absence of direct head kinematic measures during real-world falls, previous literature was reviewed to determine the appropriate head impact angle and tangential velocity. When determining anvil angle for the selected normal head impact velocities, the 20° and 25° anvil had average tangential velocities that were 0.2 m/s lower and 0.4 m/s greater than the average tangential velocity reported in literature (2.20 m/s), respectively (Table 2). While vertical motion/normal velocity is the predominant velocity vector in fall scenarios due to the minimal horizontal motion, it is important to consider the tangential component to evaluate safety helmets’ ability to mitigate rotational forces. No single anvil angle can represent all fall scenarios; therefore, it was important to select an angle that induced greater tangential velocity rather than insufficient, enabling a more effective evaluation of safety helmets’ ability to mitigate rotational acceleration. The anvil angle chosen for this study also aligns with previous modeling studies that examined head impact angles during various work-related fall scenarios. While studies by Yu et al. and Wang et al. found mean impact angles of 71°–75°, this would translate to an anvil angle of 15°–19°, which is similar to the one chosen for this study [50, 51].

**Fig. 8:**
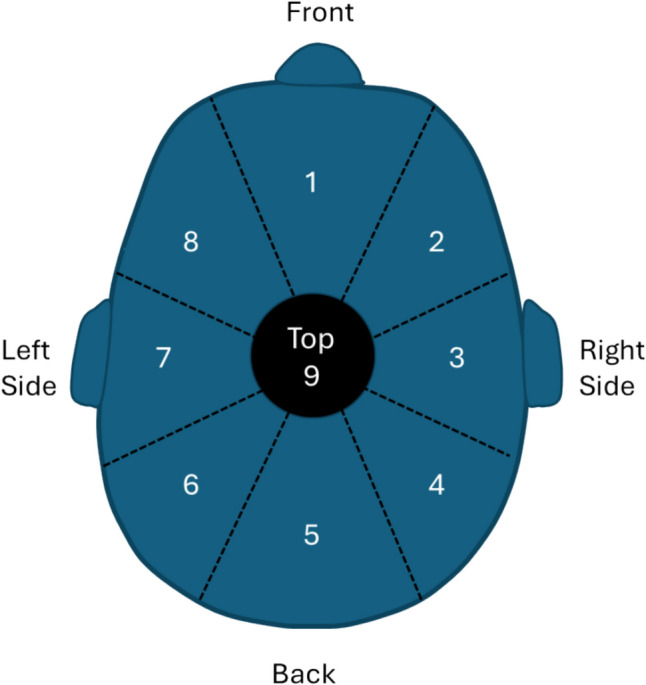
The possible impact locations outlined in the fall survey

Three impact locations for impact testing were identified through evaluating previous literature. All locations were selected to be closer to the helmet’s brim rather than the crown, as previous literature has suggested that this is a common impact location for falls not covered in standards testing [3, 34, 50]. The centered rear location was selected due to the prevalence of centered rear hits observed in both the Ferro et al. study and the simulations performed by Yu et al. [50, 52]. The rear boss location was chosen since both backwards and sideways ladder falls resulted in off-centered rear head impacts, indicating that this may be a common head impact area for multiple fall directions [52]. This is especially true when considering that ladders were the most common fall surface, as identified by the OSHA accident report search. Lastly, the front boss location was informed by the simulation work performed by Yu et al., survey responses from Proctor et al., and survey responses from the industrial head impact survey [50, 71]. Survey responses from Proctor and Rowland indicate the frontal impacts are the most common location in all industrial head impact scenarios, indicating they are a relevant location for safety helmet testing [71]. However, this study is limited as it over-generalizes impact locations and is not specific to falls. The Yu et al. study found that their high-speed forward fall resulted in an off-centered frontal impact [50]. While survey responses were limited, two out of the four responses contained impacts to the front boss location (Location 8, Fig. 8), confirming that this is a possible head impact location observed in real-world fall scenarios. When combining all this information, a front boss location was selected to evaluate the front location while accounting for the slight head rotation that is commonly observed in forward fall scenarios [102]. Although not used to determine locations, the head impact locations selected for this methodology align with the simulation work by Wang et al., as they suggest that rear and lower locations on the head were the most common during workplace-related fall accidents [51]. The locations selected for this study are limited as they rely on previous literature and generalized head impact locations. To better understand the real-world head impact locations from falls, clear videos of fall events or detailed recounts of a large sample of fall scenarios would allow for more precise head impact locations, rather than the approximate regions selected in this study.

Static friction testing showed that 80-grit sandpaper produced a higher COF against various helmet shell materials compared to concrete. Consequently, a commercial floor tile with a lower COF was considered for use in impact testing. However, the study’s findings were limited by the use of only one type of concrete, which may have influenced both the measured COF and the choice of a matching tile. In real-world settings, concrete finishes can vary widely (i.e., brushed, smooth, rough, etc.) which would affect COF values. Although the commercial tile more closely approximated the COF of the tested concrete, it resulted in significant variability in peak rotational head kinematics both within and across helmet models. The direction of rotation across helmet models also differed between locations, which has been observed in other helmet testing studies on low-friction surfaces [64, 103]. While real-world head impacts onto a low-friction surface are possible, the lack of neck constraint within the test system may be producing higher rotational head kinematics and unrealistic head rotation directions, as observed during high-speed videos of the tile impacts [64, 104, 105]. For these reasons, sandpaper was selected for further helmet testing. This study was also limited in that it did not evaluate the impact of surface stiffness. The most common impact surface identified was concrete, a stiff surface, and therefore tests were conducted on a rigid anvil. However, falls can occur on softer surfaces, which is expected to result in lower risk.

When observing the two impact energies selected for the STAR methodology (5.5 m/s and 6.8 m/s), there was a wide range of performance across the peak head kinematics, concussion risk, and skull fracture risk (Figs. 5 and 6). At the low impact speed, the average concussion risk was 65.3% across all helmet models and 56.7% if the UNLINE Standard Hard Hat was excluded, indicating that the safety helmet models tested have the potential to prevent concussions during falls representative of a 50th percentile fall height (14 ft). This is supported by the OSHA accident data, as 58.2% of falls at or below 14 ft were non-fatal, indicating that less severe head injuries such as concussions may be more prevalent at this fall height. Additionally, there was a wide range of concussion risk at the low-speed configuration, demonstrating substantial differences in helmet design and performance across safety helmet models (Fig. 6). When examining the high-speed impact condition, concussion risk was considerably higher, averaging 90.1% across all helmet models and 88.4% if the UNLINE Standard Hard Hat was excluded. All helmet models had a near 100% concussion risk at the front boss location, highlighting a weak area in several helmet designs. Because of the prevalence of skull fracture injuries from the OSHA accident data (50.2% of all injuries) and concussion risk totaling 100% during the high-speed impacts at certain locations, it was also important to evaluate skull fracture risk. Skull fracture risk during high-speed impacts averaged 72.5% across all helmet models and 68.0% when the UNLINE Standard Hard Hat was removed. The OSHA accident report analysis found that while 60% of injuries for falls above 25 ft were skull fractures, 33.8% of those falls were non-fatalities, indicating that this more severe impact condition selected for this test methodology is survivable. Although the high-speed impacts approach the upper threshold of safety helmet performance in terms of concussion prevention, helmets capable of mitigating skull fracture during high energy fall scenarios have the potential to reduce the number of real-world fatalities and catastrophic head injuries. The two impact velocities selected for testing were also within the range of a modeling study of work-related fall events that reported linear head impact velocities of 6.5 ± 2.5 m/s from fall heights of 1–5.35 m (3.3–17.6 ft) [51].

While evaluating safety helmets for concussion and skull fracture risk was important due to the prevalence of both injuries during real-world falls, it also created greater differentiation between Type I and Type II safety helmets. If only concussion incidence was used to assess safety helmets, the Type I helmet (UNLINE Standard Hard Hat) tested in this test series would have performed within the range of some of the Type II helmets (Fig. 8). However, during impact testing, the UNLINE Standard Hard Hat was the only helmet model where we could not complete all high-speed test, as it exceeded a PLA of 350 g during the low-speed trials. By incorporating skull fracture risk into the STAR equation, these differences in helmet performance across helmet types can be better observed than if just concussion risk was evaluated. Findings from other studies have agreed with this concept and found skull fractures to be a prevalent injury during falls in construction, supporting the idea that safety helmets need to be evaluated for both concussion and skull fracture risk [18, 28].

Variations in STAR score within the Type II models were also observed (Fig. 7). The two top-performing helmets (PIP Traverse MIPs and Milwaukee Tool BOLT) mitigated concussion and skull fracture risk more than the other Type II models (Honeywell Matterhorn A89 and ERB Safety Americana 360). While the top-performing helmets reported lower average peak head kinematics, the most noticeable differences between the two groups occurred when observing PLA. A possible explanation for these differences in performance may be due to design differences. The Milwaukee Tool BOLT and PIP Traverse MIPS both contain chin straps and expanded foam all throughout the helmet, whereas the ERB Safety Americana 360 and Honeywell Matterhorn A89 only have a strip of foam above the brim and a dial retention system to secure the helmet. Although only a small sample of safety helmet models were examined, these findings may indicate that design differences across Type II models may result in large variations in helmet performance.

We selected injury metrics based on the primary injury mechanisms observed in fall-related head impacts. Strain-based criteria and rapid strain estimators are promising, particularly in scenarios where rotational shear driven by velocity changes [106, 107]. However, multiple studies have shown that combined measures that incorporate linear and rotational acceleration have been reported to be more strongly associated with head injury outcomes than rotational only metrics [36, 108–110]. Falls occurring during construction activities tend to involve more linear impacts, resulting in skull fractures, thereby making linear acceleration the most pertinent factor for fracture risk assessment. Conversely, concussion risk is more accurately evaluated using combined linear and rotational measures, as multiple injury mechanisms are involved. The use of these combined and linear metrics within the STAR framework ensures consistency with previous helmet assessments. It also aligns more effectively with the fall mechanics examined, while retaining the flexibility for future benchmarking of newer criteria against fall-specific data.

This study has limitations. First, our evaluation focused on falls from height derived from the OSHA Fatality and Catastrophe Investigation Summaries database, which includes only incidents resulting in fatal or catastrophic injury. Consequently, the fall height distribution analyzed here represents this severe subset of construction accidents and does not capture the broader spectrum of nonfatal fall events. In 2022, there were 397 fatal falls to a lower level compared to approximately 19,400 non-fatal falls to lower levels [111], indicating that fatal and catastrophic events are overrepresented relative to the broader population of construction falls. However, it does represent severe but potentially survivable impact conditions in which safety helmets could meaningfully influence outcomes and complements existing helmet testing standards that primarily assess lower-energy impacts. Further, the Construction STAR evaluation system is designed to supplement existing safety standards by addressing elevated fall-related head injury risk; it does not evaluate other protective functions, such as falling object protection, electrical safety, or heat management.

Additionally, all headforms have limitations in their biofidelity, including frictional properties and controlling the fit of helmets [81, 112–114]. Third, although fracture risk is anchored to linear acceleration, our concussion risk function draws on football datasets [36, 69]; while combined linear and rotational measures are mechanistically reasonable for falls, construction-specific concussion thresholds were not evaluated. Finally, the absence of large-scale, sensor-derived field kinematics for construction helmets limited opportunities for detailed post-event reconstructions, making direct real-world validation challenging. Our methods, therefore, rely on the best available evidence from OSHA accident reports, prior literature, and controlled experimental testing. Thus, these findings should be interpreted accordingly: STAR values provide comparative performance metrics across helmets under modeled fall conditions, not exact predictions of injury incidence in the field. Despite these constraints, the approach differentiates helmet designs more meaningfully than binary pass/fail standards, offering a practical framework that can be refined as construction-specific field data and validation studies accumulate.

This study examined previous literature, analyzed accident report data, and conducted physical experimentation to identify laboratory impact conditions that can be used to evaluate safety helmets for protection against falls. The proposed methodology was then executed on five representative safety helmet models to ensure that the selected impact energy levels and locations provided a meaningful range of concussion and skull fracture risk that could be used to assess helmet performance. The low-speed impacts resulted in a wide range of concussion risk, indicating that current safety helmets have the potential to prevent concussions during falls representative of a 50th percentile fall height. While concussion risk was nearly saturated during high-speed impacts, skull fracture risk varied across helmet models, highlighting meaningful differences in their protective capabilities against skull fractures during more severe impacts. Including skull fracture risk in the STAR score created large disparities between the Type I and II models, demonstrating their differences in protection and design. Differences in STAR score across Type II models also revealed how various design features may play an important role in the safety helmet’s level of protection. Because falls account for the greatest number of fatal and non-fatal WRTBIs, safety helmets that are designed to perform well when tested under this proposed methodology have the potential to reduce the incidence rate of WRTBIs, skull fractures, and fatalities [24–27]. The Construction STAR system quantifies relative differences in helmet performance under fall-representative laboratory impacts. The STAR score generated for each helmet model enables consumers to make informed purchasing decisions when comparing safety helmets and encourages manufacturers to develop helmet designs that protect against fall scenarios.

## Data Availability

The datasets generated during and/or analyzed during the current study are available from the corresponding author on reasonable request.

## Appendix

See Fig. 8.
